# Physical activity promotion for multimorbid patients in primary care settings: a protocol for a systematic review evaluating health benefits and harms

**DOI:** 10.1186/s13643-020-01379-6

**Published:** 2020-05-13

**Authors:** Simone Schweda, Inga Krauss

**Affiliations:** 1 Medical Clinic Tuebingen, Department of Sports Medicine, Hoppe-Seyler Str. 6, 72076 Tuebingen, Germany; 2 Interfaculty Research Institute for Sports and Physical Activity, University Tuebingen, Tuebingen, Germany

**Keywords:** Multimorbidity, Physical activity, Primary care, Health, Effectiveness, Efficacy, Safety

## Abstract

**Background:**

To date multimorbidity has not received much attention in health policies, even though multiple chronic diseases put high demands on the health care system in industrial nations. Enormous costs of care and a physically, mentally, and socially reduced quality of life are common consequences of multimorbidity. Physical activity (PA) has a positive preventive and therapeutic effect on common non-communicable diseases. The objective of this study will be to evaluate the health benefits and harms of PA interventions for sedentary adults with multimorbidity in primary care settings.

**Methods:**

This is the study protocol for a systematic review. We will search PubMed, MEDLINE (Ovid), Web of Science, CINHAL, and the Cochrane Library (from inception onwards). In addition, clinical trial registers and reference lists of included studies will be searched. We will include randomized controlled trials, quasi-experimental, and non-randomized trials examining the health benefits and harms of PA interventions with or without additional lifestyle interventions for sedentary adult patients with multimorbidity (e.g., two or more chronic non-communicable diseases) in primary care. Eligible control groups will be standard care, placebo, or medications. Two reviewers will independently screen all citations, abstracts data, and full-text articles. The primary outcomes will be health-related quality of life and mortality. Secondary outcomes will include cardiovascular fitness, muscular strength and disease-specific outcomes (e.g., depression score), biomarkers as well as control of metabolic risk factors (e.g., blood pressure, HBA1c, body weight) and any adverse event. The methodological quality of the studies will be appraised using appropriate tools. If feasible, we will conduct random effects meta-analysis. Additional analyses will be conducted to explore the potential sources of heterogeneity (e.g., study design, geographical location, or type of intervention). Strength of the body of evidence will be assessed according to the Grading of Recommendations Assessment (GRADE).

**Discussion:**

This review will evaluate the evidence on health benefits and harms of PA interventions for sedentary adults with multimorbidity in primary care settings. We anticipate our findings to be of interest to patients, their families, caregivers, and healthcare professionals in selecting and conducting optimal health promotion programs. Possible implications for further research will be discussed.

**Systematic review registration:**

Open Science Framework (registration identifier: osf.io/ka8yu)

## Background

The treatment of people with multiple chronic conditions, referred to as multimorbidity [1], has not gained much attention in health politics so far. Even though, from a health economic perspective, chronic conditions belong to the most common health problems of industrial nations [2]. Multimorbidity is no longer an exception in primary care [3]. The prevalence of multimorbidity in Germany in 2008 for example was 46% in 40–85-year-olds [4] and as stated in 2013 an estimated 50 million people in the European Union (EU) live with multiple chronic conditions [5, 6]. Having multiple chronic diseases is associated with higher costs of care, more and longer hospital stays, reduced quality of life, and psychological distress [7]. Disease specific policies, focusing on one chronic disease exist in some countries such as Italy, Germany, and Netherlands [5]. In the treatment of multiple chronic conditions though single disease approaches do not seem to be appropriate. Still, policies on multimorbidity have not yet been developed [5, 8]. Many chronic conditions such as widespread diseases like Diabetes mellitus type 2 (DM type 2), obesity, cardio-vascular diseases and osteoarthritis have common risk factors. All of the named diseases are related to low-grade inflammation [9–11] that has been shown to be reduced by physical activity and exercise. Furthermore metabolic, hormonal, and cardiovascular adjustments as physiologic benefits have been documented [12]. The positive therapeutic effect of PA on several clinical conditions has been proven and is equal or even superior to drug therapy [12]. For this reason, PA has been described as a “multipill” or “polypill” [13]. Various professional societies have published training recommendations for the named individual chronic diseases [14]. Although recommendations for physical activity have a high degree of similarity for different patient groups, such recommendations do not explicitly address multimorbid patients and hardly any training recommendations for this patient group exist so far [3]. It therefore seems very promising to explicitly address multimorbid patients or patients with several risk factors for multiple chronic diseases, with an activity program that takes into account the individual characteristics of people with multiple illnesses. These interventions should take sufficient account of disease-related conditions and address personal preferences, personal habits, and the individual’s lifestyle [15]. Existing studies have shown that the combination of PA and behavioral change techniques or further lifestyle interventions for example diets in case of overweight, are most effective [14, 15]. The inclusion of behavior change techniques or other lifestyle interventions is therefore common in contemporary exercise RCT’s [16].

The objective of this study will be to evaluate the health benefits and harms of PA interventions for sedentary adults with multimorbidity in primary care settings.

## Methods

The present protocol has been registered within Open Science Framework (registration identifier: osf.io/ka8yu) and is being reported in accordance with the reporting guidance provided in the Preferred Reporting Items for Systematic Review and Meta-Analysis Protocols (PRISMA-P) statement [17, 18] (see checklist in Additional file 1). Any amendments made to this protocol will be outlined and reported in the final manuscript. The review team is multi-disciplinary and includes a reference librarian, clinician researchers, and content experts.

### Eligibility criteria

Studies will be selected according to the following criteria: participants, interventions, and comparators as well as outcome(s) of interest and study designs.

#### Participants

We will include studies conducted in sedentary adults with two or more chronic non-communicable diseases (“multimorbidity”). The focus of interest is on the following chronic conditions: cardiovascular diseases, metabolic diseases and musculoskeletal disorders, pulmonic disease, and psychological or psychosomatic disorders. All of the named diseases have a low-grade inflammation as a common risk factor [9, 19]. An increased systemic level of some cytokines and C-reactive protein characterizes low-grade inflammation [19]. Studies including people suffering one disease only or including hospitalized patients only will be excluded.

#### Interventions and comparators

We will include studies examining PA interventions in primary health care settings or public programs with or without additional psychological, nutrition, or lifestyle interventions. The intervention programs should focus on physical exercise, including diverse forms of PA promotion with or without additional interventions (e.g., behavior change techniques, lifestyle change or nutrition diary, etc.). Studies evaluating rehabilitation programs will be excluded. Eligible control group will be standard of care, placebo or other forms of therapy.

#### Outcomes of interest

The primary outcomes will be health-related quality of life (measured by a validated tool, e.g., EuroQoL 5-dimension instrument (EQ-5D) [20], Short Form (SF)-36/12 [21], or others) and mortality. Secondary outcomes will be cardiovascular fitness (VO^2^max, power output or others), muscular strength, disease-specific outcomes (e.g., Patient Health Questionnaire (PHQ) [22]), biomarkers (e.g. CRP, IL6, or others) and metabolic risk factors for the diseases of interest (e.g., blood pressure, HBA1c, body weight/BMI), physical function (e.g., Western Ontario McMaster Universities Osteroarthritis (WOMAC) [23], Knee Injury and Osteoarthritis Outcome Score (KOOS) [24], subscale physical functioning of the SF 36, or others), adherence to exercise and any adverse events.

#### Study designs

Eligible studies will be randomized controlled trials, quasi-experimental trials, and non-randomized trials, including longitudinal/prospective studies. We will exclude case reports, case series, case-control studies and single-arm cohorts. Only original studies considering humans will be considered for this review. For the initial search no language restriction will be made. Articles not published in English or German are attempted to be translated. In case no translation is possible the article will be excluded for the review.

### Information sources and search strategy

The primary source of literature will be a structured search of major electronic databases (from inception onwards): PubMed, MEDLINE (Ovid), Web of Science, CINHAL, and the Cochrane Library. The secondary source of potentially relevant material will be a search of the grey or difficult to locate literature, including clinical trial registers (such as ClinicalTrials.gov). We will perform hand-searching of the reference lists of included studies, relevant reviews, national clinical practice guidelines, or other relevant documents. The search will be conducted by the review team which includes an experienced health information specialist. The Search will include a broad range of terms and keywords related to “multimorbidity,” “exercise,” “PA,” and “primary care.” A draft search strategy for PubMed is provided in Additional file 2.

### Screening and selection process

The studies will be assessed according to the eligibility criteria and the selection process will be divided into two phases. Data will be managed transferring references to an online reference management tool (EndNote X9.2 reference manager). For further documentation Review Manager 5.3 (The Cochrane Collaboration, London, UK) will be used. Documentation of the selection process will be conducted in the management tools. As a first step duplicates of the initial search results will be removed. The initial search results will be screened by title and abstract. After comparison and agreement of the first screening process, eligible full texts will be analyzed. The screening of titles, abstracts and full texts will be hold by two independent reviewers. Any disagreement will be resolved by discussion or by including a third reviewer, if no consent can be obtained. If uncertainties persist as to the eligibility of an article, the authors may be contacted for clarification. Also, if no full text is available, the authors may be contacted. In case no full text will be received, the study will be excluded. The process will be tracked in a flow diagram according to the PRISMA design.

### Quality assessment

All of the included studies will be critically appraised by two independent reviewers to assess the methodological quality. Therefore, study-specific critical appraisal tools of the Joanna Briggs Institute will be used [25]. In case of disagreement, the results will be discussed and if needed be resolved by a third party.

Next to the methodological quality the checklists are used to determine the extent of which studies address the possibility of bias in design, conduct, and analysis. The included questions can be replied to with yes/no/unclear and not applicable. The checklist for RCT’s includes 13 questions addressing, inter alia, randomization sequence, blinding, and outcome measurements [26]. The checklist for quasi-experimental studies consists of 9 questions. The aim of the study such as possible comparators or the inclusion of a control group and statistical analyses are addressed amongst others [26]. It is not planned to weigh study results based on quality assessment or to exclude studies due to low methodological quality. But the quality assessment will be stated and considered in the discussion of the results.

### Data collection process

Two independent reviewers will perform the data extraction by use of a pre-specified data extraction sheet. This includes full participant description and study design. Also therapy characteristics such as dosage principles (type of exercise, frequency, duration, and intensity) of applied PA and PA promotion will be extracted. In this regard, efforts to take account of the individual or diseases specific elements are of particular interest. For a detailed overview, characteristics of the interventions such as the use of behavior change theories or other intervention techniques will be extracted as well. Also the methods used for evaluation will be considered. Results will be reported as mean values, standard deviations, effect sizes, and confidence intervals (if available). Any disagreements will be resolved by a third reviewer. In case data is insufficient or unavailable the authors of the studies may be conducted for clarification.

### Data synthesis

Each publication will be examined on an individual base and results will be compared if possible. A quantitative synthesis of the results is aimed for. Where data support quantitative synthesis (two or more studies which report a similar primary outcome [27]), meta-analysis will be conducted. Since clinical and epidemiological heterogeneity is expected a priori, meta-analyses will be conducted using the random effects model where appropriate. The random effects model assumes the treatment effects follow a normal distribution, considering both within-study and between study variation. Forest plots will be used to visualize pooled estimates and the extent of heterogeneity among studies. We will quantify statistical heterogeneity by estimating the variance between studies using *I*^2^ statistics. The *I*^2^ statistic is the proportion of variation in prevalence estimates that is due to genuine variation in prevalence rather than sampling (random) error. *I*^2^ statistics ranges between 0 and 100% (with values of 0–25% and 75–100% taken to indicate low and considerable heterogeneity, respectively). We will also report Tau2 and Cochran *Q* test with a *P* value of < 0.05 considered statistically significant (heterogeneity). Focus will be paid to the kind of exercise applied to the participants and the achieved effect and safety in disease related factors. Additional used intervention modules are stated and effects analyzed if achieved. Overall effect sizes including 95% confidence intervals will be generated for the studies if possible. Publication bias will be calculated using Egger’s test and funnel plots.

Subgroup analysis will be performed if adequate studies exist for interventions and outcomes. Such analysis would be conducted for the type of intervention (strength training vs. endurance training vs. combination of both) and gender. Review data analysis will be conducted by use of R in its newest release (The R Foundation) [28].

### Confidence in cumulative estimates

The strength of evidence will be assessed using the Grading of Recommendations Assessment (GRADE) [29, 30]. This will help to define the resulting evidence of the systematic review.

Any amendments to the protocol will be provided with date and description of the changes and the rationale. The table of the description will be added to the final manuscript and report of the systematic review.

## Discussion

The results of this review will help researchers and practitioners how PA promotion programs can be developed to enhance health outcomes of people with multiple chronic conditions. The demand for more and better conservative treatment options can only be fulfilled successfully with the knowledge on kind of exercise and safety of PA interventions for multimorbid patients. This analysis will also add knowledge to describe dosage principles of exercise needed for participants to be successful and able to modify their lifestyle and PA behavior. The available evidence should help to present proven health-promoting approaches with an additional focus on individualization and the underlying theoretical framework. Furthermore existing research gaps can be identified. These findings can subsequently be used to carry out further investigations.

One strength of this study is that only studies addressing patients with two or more simultaneously present diseases will be included. Interventions specifically designed for multimorbidity can take better account of the risks posed by simultaneous, multiple chronic diseases, and therefore appear more appropriate and more effective than individual disease approaches. This should lead to a broader insight in the treatment of people with multiple chronic diseases. Due to the low number of studies on multimorbidity and physical activity, a large number of heterogeneous studies could result. This might be a limitation this study has to face. In case it is not possible to perform a meta-analysis for individual studies, a meta-narrative approach will be considered to consolidate study results.

Only effective lifestyle interventions add to meaningful results in the treatment of multimorbidity and lead to an improvement in the quality of life of the patients and therefore reduce costs for the health care system. Appropriate combinations of treatment options for multimorbid patients in addition to PA will further enhance the benefits of interventions and help providers to develop and implement best practice interventions in their daily routine.

## Supplementary information

**Additional file 1.** PRISMA-P checklist.

**Additional file 2.** PubMed search strategy.

## Data Availability

Most data generated or analyzed during this study are included in this published article and its additional files. Further datasets used during the current study are available from the corresponding author on reasonable request.

## Abbreviations

CINHAL: Cumulative Index to Nursing and Allied Health Literature
DM Type 2: Diabetes mellitus type 2
EQ-5D: EuroQoL 5 dimension instrument
GRADE: Grading of Recommendations Assessment
KOOS: Knee Injury and Osteoarthritis Outcome Score
PA: Physical activity
PHQ: Patient Health Questionnaire
PRISMA: Preferred Reporting Items for Systematic Reviews and Meta-Analyses
PRISMA-P: Preferred Reporting Items for Systematic Reviews and Meta-Analyses Protocols
RCT: Randomized controlled trial
SF 36/12: Short Form 36/12
WOMAC: Western Ontario and McMaster Universities Osteoarthritis
