# Supplementary material for: Physical activity promotion for multimorbid patients in primary care settings: a protocol for a systematic review evaluating health benefits and harms
Source: Syst Rev. 2020 May 13;9:110. doi: 10.1186/s13643-020-01379-6 (PMC7222306; doi:10.1186/s13643-020-01379-6)
Supplement: Supplementary file 2 — Additional file 2. PubMed search strategy. [file 13643_2020_1379_MOESM2_ESM.docx]

**File 2:**

Search strategy for PubMed

#1 "Musculoskeletal Diseases"[Mesh:noExp] OR Musculoskeletal Disease*[tiab] OR Musculoskeletal Disorder*[tiab] OR Orthopedic Disorder*[tiab] (17839)

#2 "Arthritis, Rheumatoid"[Mesh] OR Rheumatoid Arthritis[tiab] (142192)

#3 "Osteoarthritis"[Mesh] OR Osteoarthritides[tiab] OR Osteoarthros*[tiab] (60440)

#4 "Metabolic Diseases"[Mesh:noExp] OR Metabolic Disease*[tiab] (27692)

#5 "Diabetes Mellitus"[Mesh] OR Diabetes Mellitus[tiab] OR Diabetes Insipidus[tiab] OR Glucose Intolerance[tiab] OR Gastroparesis[tiab] (487105)

#6 "Hyperlipidemias"[Mesh] OR Hyperlipidemia*[tiab] OR hyperlipemia*[tiab] OR Hypercholesterolemia*[tiab] OR High Cholesterol Level*[tiab] OR Elevated Cholesterol[tiab] OR Hypercholesteremia[tiab] (88923)

#7 "Hypertriglyceridemia"[Mesh] OR hypertriglyceridemia*[tiab] (13818)

#8 "Osteoporosis"[Mesh] OR osteoporosis[tiab] OR osteoporoses[tiab] (82760)

#9 "Heart Diseases"[Mesh] OR Heart Disease*[tiab] OR Heart disorder*[tiab] OR heart failure*[tiab] OR cardiac failure*[tiab] OR Cardiac Disease*[tiab] OR cardiac disorder*[tiab] OR cardiovascular disease*[tiab] OR cardiovascular disorder*[tiab] OR cardiovascular failure*[tiab] OR coronary disorder*[tiab] OR coronary disease*[tiab] OR coronary failure[tiab] OR Cardiac Dysrhythmia*[tiab] OR Cardiac Arrhythmia*[tiab] OR Arrhythmia*[tiab] OR Arrythmia*[tiab] (1332247)

#10 "Hypertension"[Mesh] OR High Blood Pressure*[tiab] OR hypertension[tiab] (454111)

#11 "Asthma"[Mesh] OR Asthma*[tiab] OR Bronchial Asthma[tiab] (171447)

#12 "Pulmonary Disease, Chronic Obstructive"[Mesh] OR COPD[tiab] OR Chronic Obstructive Pulmonary Disease[tiab] OR COAD[tiab] OR Chronic Obstructive Airway Disease*[tiab] OR Chronic Obstructive Lung Disease*[tiab] OR Chronic Airflow Obstruction[tiab] OR pulmonary disease*[tiab] OR pulmonary disorder*[tiab] (99607)

#13 #1 OR #2 OR #3 OR #4 OR #5 OR #6 OR #7 OR #8 OR #9 OR #10 OR #11 OR #12 (2628628)

#14 "Mental Disorders"[Mesh] OR Mental Disorder*[tiab] OR Psychiatric Diagnosis[tiab] OR Behavior Disorder*[tiab] OR Mental Disease*[tiab] OR anxiety disease*[tiab] OR anxiety disorder*[tiab] OR mood disease*[tiab] OR mood disorder*[tiab] OR psychological disease*[tiab] OR psychological disorder*[tiab] OR Depression*[tiab] OR Depressive Symptom*[tiab] (1403160)

#15 #13 OR #14 (3944845)

#16 (co exist*[tiab] OR multiple[tiab] OR Co occur[tiab] OR Cooccur[tiab] OR Coexist[tiab] OR chronic[tiab]) AND (disease*[tiab] OR disorder*[tiab] OR illness[tiab] OR condition*[tiab] OR syndrome*[tiab] OR symptom*[tiab]) (1062001)

#17 #15 OR #16 (4651677)

#18 "Primary Health Care"[Mesh:noExp] OR Primary Healthcare[tiab] OR Primary Care[tiab] (141088)

#19 ((basic medicine[tiab] OR health[tiab]) AND (care[tiab] OR service*[tiab] OR program*[tiab] OR intervention*[tiab])) (881701)

#20 ((citizen center*[tiab] OR patient center*[tiab]) AND care[tiab]) (11091)

#21 ((community*[tiab] OR primary*[tiab] OR general*[tiab]) AND (care[tiab] OR setting*[tiab] OR provider*[tiab] OR physician*[tiab] OR service*[tiab] OR practitioner*[tiab])) (665777)

#22 #18 OR #19 OR #20 OR #21 (1305843)

#23 "Rehabilitation"[Mesh:noExp] OR rehabilitation[tiab] (160674)

#24 "Home Care Services"[Mesh] OR Home Care Service*[tiab] OR Domiciliary Care[tiab] OR Home Nursing[tiab] OR hospital based care*[tiab] (47362)

#25 "Hospitalization"[Mesh] (223171)

#26 "Hospice Care"[Mesh] (6143)

#27 #23 OR #24 OR #25 OR #26 (420892)

#28 #22 NOT #27 (1210126)

#29 (Exercise*[tw] OR Physical Activit*[tiab] OR Training[tiab] OR "Sports"[tw] OR Athletic*[tiab]) AND (program*[tiab] OR intervention*[tiab] OR therapy[tiab] OR therapies[tiab] OR meeting[tiab] OR session[tiab] OR strategy[tiab] OR Workshop*[tiab]) (280326)

#30 ((Endurance training*[tiab] OR aerobic*[tiab] OR stamina[tiab] OR perseverance training*[tiab] OR cardiovascular*[tiab]) AND (training*[tiab] OR Exercise*[tiab] OR workout*[tiab] OR program*[tiab] OR intervention*[tiab] OR therapy[tiab] OR therapies[tiab] OR meeting[tiab] OR session[tiab] OR strategy[tiab] OR workshop*[tiab])) (158323)

#31 ((strength training*[tiab] OR Weight-Lifting[tiab] OR Weight-Bearing[tiab] OR Strengthening Program*[tiab] OR power[tiab]) AND (training*[tiab] OR Exercise*[tiab] OR workout*[tiab] OR program*[tiab] OR intervention*[tiab] OR therapy[tiab] OR therapies[tiab] OR meeting[tiab] OR session[tiab] OR strategy[tiab] OR workshop*[tiab])) (64914)

#32 "Gymnastics"[Mesh] OR gymnastic*[tiab] (3470)

#33 "Physical Education and Training"[Mesh] OR physical education[tiab] (15457)

#34 "Tai Ji"[Mesh] OR Tai chi[tiab] OR Tai-ji[tiab] OR Taiji[tiab] OR "T'ai Chi" OR chi gong[tiab] (1782)

#35 "Yoga"[Mesh] OR yoga[tiab] (4749)

#36 "Ergometry"[Mesh] OR Ergometr*[tiab] OR Ergometer[tiab] (71189)

#37 "Bicycling"[tw] OR cycling[tiab] (63111)

#38 "Walking"[tw] (76657)

#39 "Running"[tw] OR jogging[tiab] OR hiking[tiab] (65459)

#40 "Swimming"[tw] OR aqua fitness[tiab] (34267)

#41 #29 OR #30 OR #31 OR #32 OR #33 OR #34 OR #35 OR #36 OR #37 OR #38 OR #39 OR #40 (714240)

#42 ((Psychological*[tiab] OR behaviour[tiab] OR attitude[tiab]) AND (technique*[tiab] OR modification*[tiab] OR intervention[tiab])) (58848)

#43 (("Life Style"[Mesh:noExp] OR Life Style*[tiab] OR Lifestyle*[tiab]) AND (technique*[tiab] OR modification*[tiab] OR intervention*[tiab])) (34884)

#44 "Diet"[Mesh] OR diet[tiab] OR diets[tiab] OR nutrition[tiab] OR food*[tiab] OR alimentari*[tiab] (928627)

#45 #42 OR #43 OR #44 (1004730)

#46 Clinical[tiab] AND trial[tiab] (252585)

#47 randomized controlled trial [publication Type] (486834)

#48 randomized[tiab] AND controlled[tiab] AND trial[tiab] (128078)

#49 controlled clinical trial[publication Type] (575166)

#50 randomized[tiab] (486842)

#51 randomly[tiab] (315970)

#52 trial[tiab] (557439)

#53 groups[tiab] (1965481)

#54 "Double-Blind Method"[Mesh] OR Double Blind Method[tiab] OR Double Blind Methods[tiab] OR Double Masked Method[tiab] OR Double Masked Methods[tiab] OR Double Masked Stud*[tiab] OR Double Blind Stud*[tiab] (158224)

#55 (double[tiab] OR single[tiab] OR triple[tiab] OR treble[tiab]) AND blind[tiab] (150458)

#56 "Random Allocation"[Mesh] OR Random Allocation[tiab] OR Randomization[tiab] (125802)

#57 "Single Blind Method"[Mesh] OR Single Blind Method[tiab] OR Single Masked Method[tiab] OR Single Blind Methods[tiab] OR Single Masked Methods[tiab] OR Single Blind Stud*[tiab] OR Single Masked Stud*[tiab] (29161)

#58 quasi experiment[tiab] OR quasiexperiment*[tiab] (1017)

#59 (("Longitudinal Studies"[Mesh] OR longitudinal[tiab]) AND ("Prospective Studies"[Mesh] OR prospective[tiab])) AND (study[tiab] OR studies[tiab] OR design[tiab] OR designs[tiab] OR trial[tiab] OR trials[tiab]) (37433)

#60 ("Adolescent"[Mesh] OR "Child"[Mesh] OR "Infant"[Mesh]) NOT "Adult"[Mesh] (1813296)

#61 "Animals"[Mesh] NOT "Humans"[Mesh] (4603389)

#62 #46 OR #47 OR #48 OR #49 OR #50 OR #51 OR #52 OR #53 OR #54 OR #55 OR #56 OR #57 OR #58 OR #59 (2937821)

#63 #62 NOT #60 (2734679)

#64 #63 NOT #61 (2285893)

#65 "Comorbidity"[Mesh:noExp] OR Comorbid*[tiab] OR co morbid*[tiab] (222969)

#66 "Multimorbidity"[Mesh:noExp] OR Multimorbid*[tiab] (4161)

#67 "Chronic Disease"[Mesh:noExp] OR Chronic Disease*[tiab] OR Chronic Illness*[tiab] OR Chronically Ill[tiab] OR Chronic Disorder*[tiab] OR chronic medication[tiab] OR chronic syndrome*[tiab] OR chronic symptom*[tiab] OR chronic condition*[tiab] OR (chronic[tiab] AND health[tiab]) (400802)

#68 "Multiple Chronic Conditions"[Mesh] OR Mulitple Chronic Condition*[tiab] OR Multiple Chronic Illness*[tiab] OR Multiple Chronic Medical Condition*[tiab] OR Multiple Chronic Medical Problem*[tiab] OR Multiple Morbidit*[tiab] OR Multiple Chronic Disease*[tiab] OR Multiple Chronic Health Conditions[tiab] (1255)

#69 Multidisease[tiab] OR multi disease*[tiab] OR multiple disease*[tiab] OR multiple ill[tiab] OR multiple illness*[tiab] OR multiple disorder*[tiab] OR multiple condition*[tiab] OR multiple syndrome*[tiab] (13019)

#70 #65 OR #66 OR #67 OR #68 OR #69 (618230)

#71 #41 AND #45 (71273)

#72 #41 OR #71 (714240)

#73 #17 OR #28 OR #64 OR #70 OR #72 (7502165)

#74 #17 AND #28 AND #64 AND #70 AND #72 (3864)
